# Bilateral Adrenal Hemorrhage Heralds Bronchogenic Carcinoma

**DOI:** 10.7759/cureus.52109

**Published:** 2024-01-11

**Authors:** Faysal Azani, Abdur Rehman Alozai, Jimmy William, Jayant Sharma

**Affiliations:** 1 Cardiology, Sligo University Hospital, Sligo, IRL; 2 Internal Medicine, Royal Preston Hospital, Preston, GBR; 3 Internal Medicine, Ziryab Research Group, Khartoum, SDN; 4 Hematology, Sligo University Hospital, Sligo, IRL; 5 Internal Medicine, Midland Regional Hospital Portlaoise, Portlaoise, IRL

**Keywords:** hemorrhage, addison's disease, adrenal metastasis, lung cancer, nsclc

## Abstract

This case study delves into the unusual presentation of non-small cell lung carcinoma (NSCLC), where bilateral hemorrhagic adrenal metastasis served as the primary indication of an underlying malignancy. Our patient, a 58-year-old male, sought medical attention due to acute abdominal pain and lower back discomfort, leading to an in-depth diagnostic exploration. Radiological examinations revealed bilateral adrenal masses exhibiting hemorrhagic characteristics, a distinctive feature not commonly associated with NSCLC. The subsequent biopsy and histopathological analysis definitively identified metastatic NSCLC as the culprit. The uniqueness of this case lies in the bilateral nature of the metastasis and the presence of hemorrhagic elements, challenging traditional diagnostic expectations.

This report emphasizes the necessity for a nuanced approach to diagnostic investigations when confronted with atypical presentations, especially considering the rarity of bilateral involvement and hemorrhagic features in adrenal metastases from NSCLC. It highlights the importance of interdisciplinary collaboration between radiologists, pathologists, and oncologists to ensure accurate and timely diagnosis. The overarching significance of this case extends beyond its rarity; it underscores the imperative for healthcare practitioners to broaden their diagnostic considerations in the absence of conventional symptoms. By presenting this distinctive case, we contribute to the evolving understanding of the diverse clinical manifestations of NSCLC, advocating for heightened vigilance and comprehensive diagnostic approaches in the pursuit of early intervention and optimal patient care.

## Introduction

Adrenal metastasis is relatively common in lung cancer, affecting up to 40% of cases [1]. Typically, these metastases are unilateral and asymptomatic, often detected during lung cancer staging. Bilateral adrenal metastasis, found in only 3% of initial lung cancer diagnoses, is rare [2]. Even rarer is the occurrence of bilateral adrenal hemorrhagic metastasis as the initial presentation of non-small cell lung carcinoma (NSCLC) lung, with very few reported cases [1]. A literature review by Marti et al. of 133 cases of hemorrhagic adrenal masses showed that only 6.8% were secondary to adrenal metastasis, with pheochromocytoma and pseudocysts being more frequent causes [3]. This case highlights the importance of comprehensive investigations when patients present with bilateral adrenal hemorrhage. NSCLC of the lung should be considered as a potential diagnosis, even if there are no apparent signs of lung malignancy on the initial CT scan.

## Case presentation

A middle-aged man in his 50s with a history of hypertension and chronic atrial fibrillation, occasional alcohol consumption, and a significant smoking history presented to the Emergency Department with severe back pain in the lower chest and lumbar regions. He had no recent trauma or traveled to tuberculosis-endemic regions, and his family history revealed no chronic illnesses (including tuberculosis) or clotting disorders. Although the patient was vitally stable, he appeared unwell with symptoms of diaphoresis, clamminess, and tachycardia, along with a blood pressure reading of 100/70. Clinical examination revealed tenderness in the paravertebral areas but was otherwise unremarkable. Lab results showed mild neutrophilic leukocytosis, an elevated international normalized ratio of 1.3, and increased D-dimers (Table 1).

**Table 1:** Laboratory investigations * Positive Short Synacthen test FBC: Full blood count; MCV: Mean corpuscular volume; APTT: Activated partial thromboplastin time; INR: International normalized ratio; CRP: C-reactive protein; ALT: Alanine aminotransferase

Lab investigation		Result	Reference range
FBC	Hemoglobin	14.1 g/dL	(13-14.2 g/dL)
	MCV	95.9 fL	(80-100 fL)
	Leukocytes	11.13 x 10^9^/L	(4-10 x 10^9^/L)
	Neutrophils	8.29 x 10^9^/L	(2-7 x 10^9^/L)
	Platelets	238 x 10^9^/L	(140-400 x 10^9^/L)
	Eosinophils	0.10 x 10^9^/L	(0.02-0.50 x 10^9^ /L)
	Basophils	0.08 x 10^9^/L	(0.02-0.10 x 10^9^ /L)
	Monocytes	0.96 x 10^9^/L	(0.20-1.00 x 10^9^/L)
	Lymphocytes	1.70 x 10^9^/L	(1-4 x 10^9^/L)
Coagulation profile	Prothrombin time	15.5 seconds	(11.5-15.5)
	APTT	32 seconds	(27.0-37.0)
	INR	1.1	
	D-dimers	1832 ng/ml	(0.0-500)
Renal function Tests	Serum sodium	141 mmol/L	(133-145 mmol/L)
	Serum potassium	4.4 mmol/L	(3.5-5.1 mmol/L)
	Serum creatinine	75 umol/L	(62-106 umol/L)
	Serum urea	4.6 mmol/L	(1.70-8.30 mmol/L)
Bone profile	Calcium	2.44 mmol/L	(2.15-2.55 mmol/L)
Others	Glucose	5.7 mmol /L	(3.50-7.80 mmol/L)
	CRP	3 mg/L	(0.20-5.0)
	Albumin	44 g/L	(35-52 g/L)
	ALT	11 U/L	(8.0-41 U/L)
Adrenal status	Random cortisol	150 nm/l	(185-624)
Short Synacthen test*	0-hour cortisol	161 nmol/L	(185-624)
	1-hour cortisol	271 nmol/L	(<276)
Autoimmune workup	Antinuclear antibodies	Positive (>1:160)	Negative
	Smooth muscle antibodies	Negative	Negative
	Mitochondrial antibodies	Negative	Negative
	Parietal cell antibodies	Negative	Negative
	DNA antibodies	Negative	Negative
	Antiadrenal antibodies	Negative	Negative

**Figure 1:**
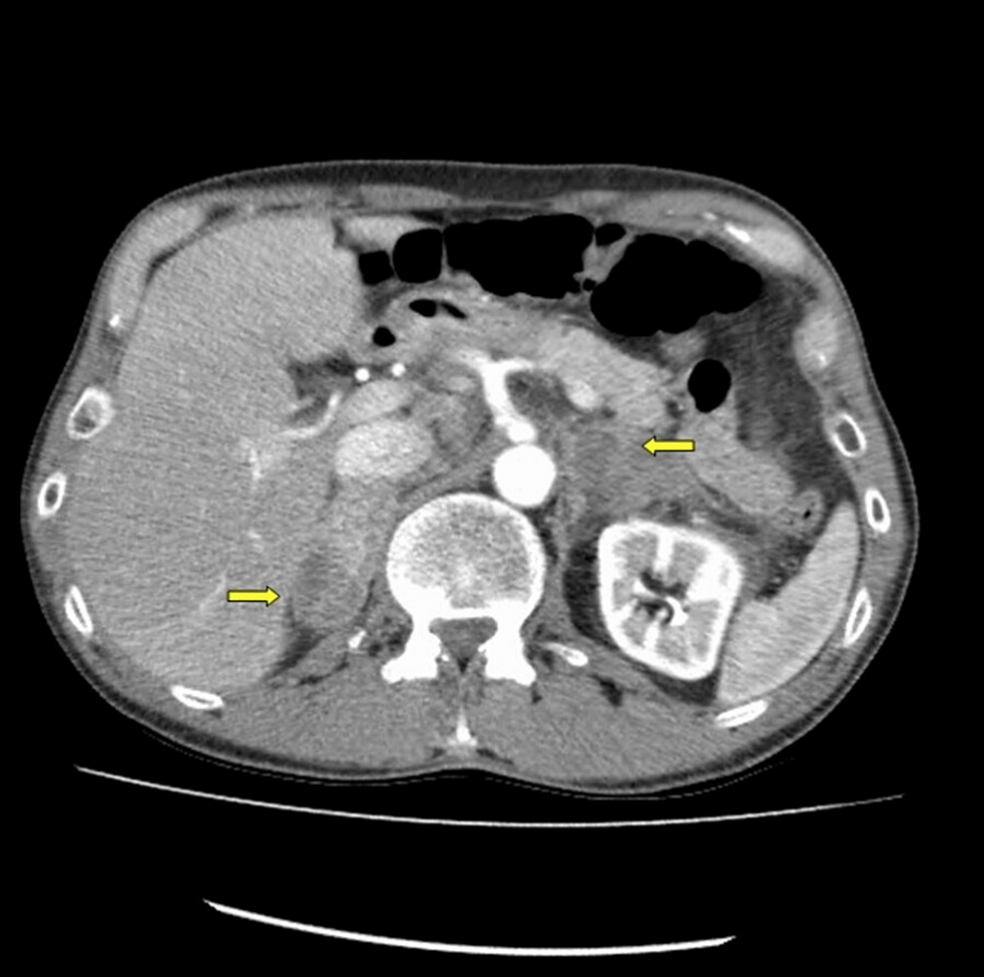
CT abdomen with contrast: bilaterally enlarged adrenals with hemorrhage and periadrenal infiltration (yellow arrows)

However, his renal and hepatic profiles, serum amylase, arterial blood gases, ECG, and chest radiograph were normal. A high-resolution CT scan of the chest ruled out pulmonary embolism but revealed significant bilateral adrenal enlargement due to spontaneous bilateral adrenal hemorrhages (Figure 1).

**Figure 2:**
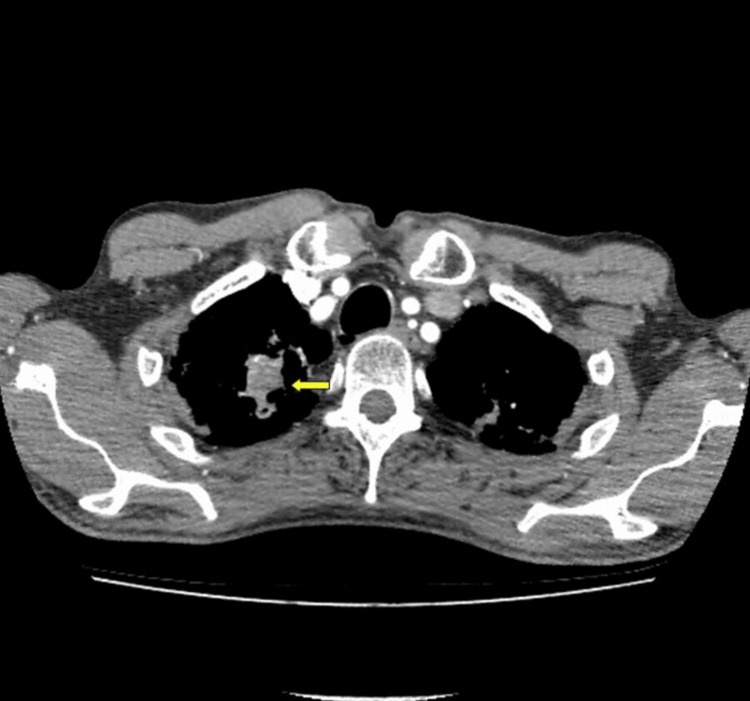
Primary pulmonary lesion on the right upper lobe (yellow arrow)

The patient's pain was only partially relieved by opiates, and his persistent tachycardia prompted an adrenal function evaluation, which revealed low cortisol levels. An urgent Short Synacthen test confirmed adrenal insufficiency, and the patient was initiated on IV hydrocortisone replacement therapy. Extensive work-up, including testing for antiphospholipid biomarkers and adrenal antibodies, did not reveal an underlying cause for the adrenal hemorrhages. He was discharged on oral hydrocortisone and fludrocortisone. Subsequent imaging showed a pulmonary mass in the right upper lobe, suggesting malignancy with metastases (Figure 2).

**Figure 3:**
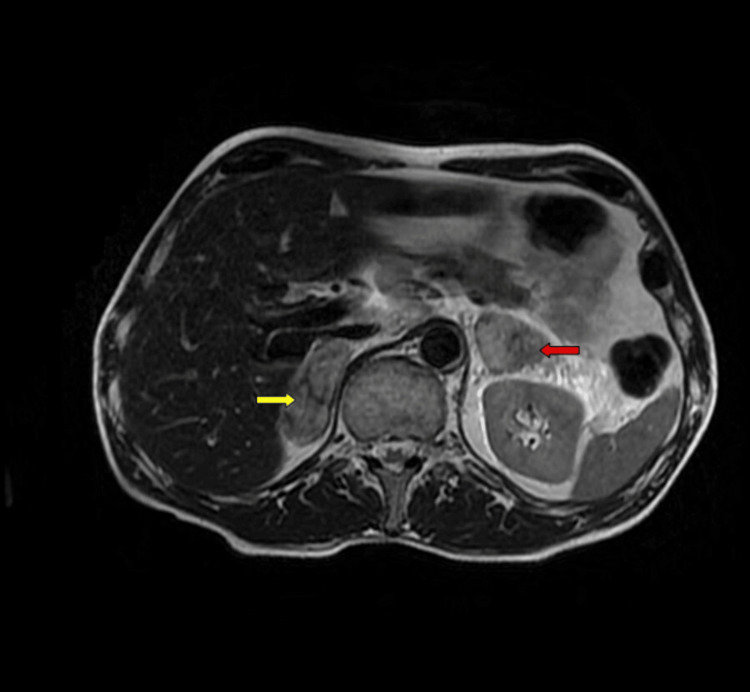
T2 MR imaging with contrast: mixed enhancement and non-enhanced center, particularly in the left adrenal gland (yellow and red arrows)

Furthermore, the MRI of the adrenals showed a resolving hemorrhage with probable metastatic lesions (Figure 3).

Bronchoscopy and transbronchial needle aspiration confirmed NSCLC with an adenocarcinoma subtype, given it showed TTF1 positivity and p63 negativity on the immune histochemical profile. The tumor was inoperable at the time of diagnosis. The patient was referred to an oncologist with endocrinology follow-up for adrenal insufficiency. He underwent chemotherapy and responded well to treatment, in addition to palliative care to manage symptoms.

## Discussion

Adrenal hemorrhage is a rare yet life-threatening condition often associated with metastatic malignancies [1]. It can lead to acute adrenal insufficiency, with adrenal metastasis prevalence ranging from 20% to 45%, depending on the primary tumor's origin [2]. Bilateral adrenal hemorrhage is exceptionally rare, estimated at an incidence of 0.14% to 1.8% [4]. Symptoms of adrenal hemorrhage can be nonspecific and may include nausea, vomiting, sudden abdominal, flank, and chest pain, tachycardia, fever, hypotension, and shock [1]. Notably, adrenal insufficiency symptoms may not manifest unless approximately 90% of adrenal function is compromised [5]. The exact pathophysiology of adrenal hemorrhage remains a subject of ongoing research [6]. Understanding the anatomy of the adrenal gland is crucial to grasp this phenomenon. While it receives a rich arterial blood supply, it has limited venous drainage, leading to vascular congestion. Proposed mechanisms include the development of a prothrombotic state, with malignancies causing thrombosis in adrenal veins, elevating vascular pressure, and causing hemorrhage [6].

Additionally, the adrenal vessels are susceptible to vasoconstriction under various conditions, contributing to hemorrhage [7]. Imaging techniques, such as CT scans and MRI, are pivotal for diagnosing adrenal hemorrhage. The key diagnostic criteria include a hyperdense lesion on non-contrast CT scans or specific MRI signal patterns [4]. However, distinguishing adrenal hemorrhage from other adrenal conditions, such as adenomas, can be challenging. Adrenal hemorrhage can result from various causes, including metastatic disease, leukemia, lymphoma, amyloidosis, myocardial infarction, granulomatous infections, pheochromocytoma, adrenal adenoma, and adrenal carcinoma [3]. Notably, subtle changes in lung parenchyma may indicate underlying malignancies, such as NSCLC, which can atypically present with bilateral adrenal hemorrhage [1].

Treatment approaches for adrenal hemorrhage remain diverse due to its rarity, with no established consensus [1]. Generally, conservative management involves fluid resuscitation, blood transfusions, and close monitoring. In select cases, like severe adrenal hemorrhage, interventions like transcatheter embolization or adrenalectomy may be considered [1]. Early detection of adrenal insufficiency is crucial, as nearly 50% of patients with bilateral adrenal hemorrhage may eventually develop adrenal insufficiency, elevating the risk of mortality [7]. For patients with extensive bilateral adrenal metastasis, close endocrinological evaluation is vital, even if initial tests appear normal [8]. While the typical presentation of NSCLC primarily includes symptoms like cough, hemoptysis, and worsening dyspnea, this case sheds light on a unique scenario wherein NSCLC presented with abdominal pain attributed to hemorrhagic adrenal metastasis [9].

## Conclusions

This case highlights the rarity of bilateral adrenal hemorrhage as the initial presentation of non-small cell lung cancer. Timely and thorough investigations, including advanced imaging and bronchoscopic evaluation, played a crucial role in diagnosing the underlying malignancy. Early recognition of adrenal insufficiency and prompt steroid replacement was pivotal in achieving a favorable outcome. The report underscores the importance of a comprehensive diagnostic approach in cases of bilateral adrenal hemorrhage, shedding light on its potential association with NSCLC.
